# New Medical Journals

**Published:** 1867-10

**Authors:** 


### *New Medical Journals.*

A new medical journal is announced as forthcoming in San Francisco. St. Louis also announces a monthly—hight, *The Humboldt Medical Archives*, to be edited by A. Hammer, M.D., and Montrose A. Pallen, M.D. $3.00 in advance; $4.00 after six months; $5.00 if after one year, a scale of increase for non-payment in advance which would startle the delinquent patrons of the present paper. The second No. of Dr. Hammond's *Quarterly Journal of Psychological Science* comes to hand delightfully rich in typographical art, and better still in its excellence of matter. The old *Medical Gazette*, a name sacred to the memory of our venerated friend, Dr. D. M. Reese, comes to us in a new dress as a weekly, under the publishing conduct of A. Simpson & Co., New York, the well-known publishers, with Lindsay and Blakiston as the Philadelphia sponsors. No editorial name is given, but, from appearances, it is intended to mingle the *utile cum dulce*, as the manner of some others we wot of is—the distant insinuation being, that there is to be enough of medicine and surgery in each number to float the advertisements. This is a pretty well understood New York and Philadelphia dodge, indulged in by several of the great publishing houses, occasionally, at the expense of legitimate medical journals. A medical journal should have some paternity aside from a mere advertising agent, in order to secure the confidence of the profession. But, meanwhile, greeting to all working colaborers.

